# Using Protein Homology Models for Structure-Based Studies: Approaches to Model Refinement

**DOI:** 10.1100/tsw.2006.250

**Published:** 2006-12-06

**Authors:** V. Kairys, M.K. Gilson, Miguel Xavier Fernandes

**Affiliations:** ^1^ Centro de Química da Madeira, Departamento de Química da Universidade da Madeira, Campus da Penteada, 9000-390 Funchal, Portugal; ^2^ Center for Advanced Research in Biotechnology, 9600 Gudelsky Drive, Rockville, MD 20850, USA

**Keywords:** homology modeling, docking, drug design, molecular dynamics

## Abstract

Homology modeling is a computational methodology to assign a 3-D structure to a target protein when experimental data are not available. The methodology uses another protein with a known structure that shares some sequence identity with the target as a template. The crudest approach is to thread the target protein backbone atoms over the backbone atoms of the template protein, but necessary refinement methods are needed to produce realistic models. In this mini-review anchored within the scope of drug design, we show the validity of using homology models of proteins in the discovery of binders for potential therapeutic targets. We also report several different approaches to homology model refinement, going from very simple to the most elaborate. Results show that refinement approaches are system dependent and that more elaborate methodologies do not always correlate with better performances from built homology models.

## INTRODUCTION

Structure-based methods are very important for several areas of computational chemistry, such as drug design, assignment of unknown protein functions, studies of reaction mechanisms, etc.

Recently, we have witnessed an explosive growth in the number of available protein sequences due to the advance in genomics. However, this vast amount of information was not accompanied by the experimental determination of proteins' 3-D structure; therefore, computational modeling methods are useful tools when experimental information is lacking. Having the structure of a protein, either experimental or modeled, has enormous significance in order to understand its function and interaction with substrate and drugs, and interactions with other proteins. The most frequently used computational method to assign the 3-D structure of proteins is homology modeling (sometimes referred to as comparative modeling). Homology modeling predicts the 3-D structure of a protein for which only the sequence is known (called target protein) using another protein with a known structure (called template). Target and template proteins must share some sequence identity (generally >30%) for the method to be effective. After proper alignment of sequences, the target protein backbone atoms are placed in identical positions to those of the template protein backbone atoms, and subsequent efforts place side chain atoms, remove clashes, and eventually minimize the energy of the modeled target protein. There has been a considerable effort put into creation of realistic homology models of proteins and it has been extensively discussed in the literature[1,2,3]. As Critical Assessment of protein Structure Prediction (CASP) experiments show, there is still much room for improvement[4,5].

In spite of inaccuracies of homology models, it has been shown that they can be used successfully in structure-based drug discovery. A rapid increase in the use of homology models for structure-based drug discovery (Fig. 1) has been seen in recent years, and this review attempts to summarize advances in the field. We will restrict our attention to the docking of small molecules into homology models, but even with this restriction, the areas of application still cover a broad range of systems (see Table 1 in Supplementary Material) and approaches. Docking is a well-established tool of structure-based drug design that tries to predict if a ligand (generally a small molecule) will bind to a receptor (generally a protein) and form a stable complex. Molecular docking has been extensively reviewed elsewhere[6,7,8].

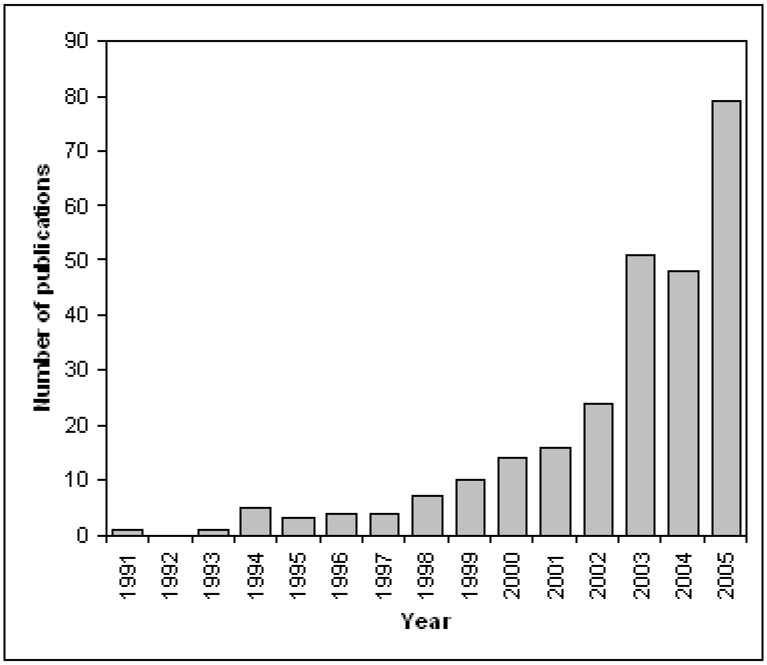

**FIGURE 1.** A crude estimate of publications that reports docking into homology models (excluding protein-protein docking). The figure was generated after searching PubMed using the keywords "docking" and "homology", and analyzing the hits.

After a hypothetical 3-D homology model is built, it may still contain inaccuracies and errors that may interfere with ligand placement inside the binding site. In this regard, optimization of the binding site with (or without) the binder can be roughly divided into several categories[9]: (1) use without further optimization or with simple optimization to remove clashes, (2) use several alternative receptor structures (an ensemble) for docking, (3) ligand-directed optimization of binding pocket, (4) partial optimization and, (5) molecular dynamics (MD) or related approaches. In the next section, we discuss the usefulness of homology models by comparing the results with those obtained using experimental structures. In the following section, we will describe some recent applications that use different approaches to homology model refinement and look into the relevant applications.

## VALIDATION OF HOMOLOGY MODEL USE

Even though we will see later that homology models can be used for drug discovery successfully, the comparison between the performances of homology models vs. experimental 3-D structures is an interesting issue. In several papers, it was shown that homology models in virtual screenings generally perform somewhat worse compared to the target proteins, but they are still able to enrich the screening results.

### Validation Based on Enrichment Analysis

In an extensive study, McGovern and Shoichet analyzed how results of virtual screenings are dependent on the accuracy of the binding site that decreases when going from crystal structure to homology models[10]. Results of screenings of compound databases were compared for the holo (ligand-bound), apo, and homology-modeled structures of ten different enzyme-binding sites. Their results suggested that the performance of the docking calculation is affected by the particular representation of the receptor used in the screening, and that the holo structure is the one most likely to yield the best discrimination between known ligands and decoy molecules, but important exceptions to this rule also emerge from this study. Although each of the holo, apo, and modeled conformations led to enrichment of known ligands in all systems, the enrichment did not always rise to a level judged to be sufficient to justify the effort of a docking screen. In this context, enrichment means the ratio of the number of known binders picked using a certain method over the number of known binders expected to be picked by random selection. If a 20-fold enrichment of known ligands is taken as a rough guideline for what might be enough to justify a docking screen, the holo conformation of the enzyme met this criterion in eight of ten cases, whereas the apo conformation met this criterion in only two cases, and the modeled conformation in three.

In the study by Diller and Li[11], homology models of six kinases were built to see if they could distinguish between known inhibitors and decoy compounds when combined with high-throughput docking. For comparison, four X-ray structures were also included in this analysis. In five of the six cases, the models were able to select the inhibitors five times over random. In the sixth case, the homology model was unable to distinguish between inhibitors and decoy compounds. In this case, a model built with an alternate template was able to distinguish inhibitors from decoy at a rate comparable to the other five models. The performance of homology models was comparable to X-ray structures and, in one case, perceptibly quite better. One of the conclusions regards the importance of template choice used for building of homology models and also the choice of X-ray structures to be used in screening (when they are available).

Oshiro et al. compared enrichments of virtual screenings with known binders by docking into CDK2 and factor VIIa homologs[12]. When the sequence identity between model and template near the binding site area was greater than approximately 50%, roughly five times more active compounds were identified than would be found randomly. This performance was comparable to docking to crystal structures.

Schapira et al. constructed a model of the inactive conformation of human retinoic acid receptor-α (RARα) by using information derived from antagonist-bound estrogen receptor-α and applied a computer-based virtual screening algorithm to identify antagonists[13]. Screening was performed against a database of known ligands and also against a large commercially available compound database, which led them to discover novel agonists and antagonists. To discover why some known ligands were missed, a docking simulation was carried out with a full atom representation of the receptor, with a Monte Carlo energy minimization of the complex, with both flexible-ligand and flexible-receptor side chains. The conformation of several receptor side chains was modified during the docking simulation to accommodate the size of the ligand, and this solution would not have been found with rigid side chains. The optimized receptor structure was used for screening, and the updated structure totally eliminated the presence of both false positives and false negatives (all RAR ligands and only RAR ligands were selected).

Kairys et al. analyzed performance of virtual screenings into homology models of carboxypeptidase A, factor Xa, PPARα, CDK2, and acetylcholinesterase, and compared the data with screenings against the X-ray structures of the targets[14]. A central result of their research was that docking into homology models frequently yields enrichments of known binders as good as docking into the actual target protein. Interestingly, however, standard measures of the similarity (% of sequence identity, Z-score, E-score) between template and target protein showed little correlation with the effectiveness of the screening calculations for the built homology models, and docking to the template itself sometimes can be as successful as docking into the corresponding homology model. Treating side chains as mobile produced modest improvement in the results.

## Validation Based on Structural Analysis

While previously analyzed studies demonstrated the capability of homology models to enrich the virtual screening results, it is of interest to analyze how homology models refined using different methods differ from the experimentally determined structure, and how these differences influence docking results. The papers analyzed below were emblematic because, in all the cases mentioned, comparison was enabled by the availability of the crystal structure in the middle or the end of the studies.

Marhefka et al. modeled human androgen receptor (hAR), which was refined using unrestrained multiple MD simulations in explicit solvent[15]. The obtained structure was compared with the experimental hAR structure because the latter was published during the research. Main chain atoms differ by 1.2 Å RMSD between the model and the crystal structure. The H-bonding patterns are different between the X-ray and MD structures, which can be explained by differences in bound ligands. In addition, an unoccupied pocket is observed in the crystal structure. Nevertheless, the quality of the model was enough so that analysis of amino acid mutations within the hAR-binding pocket that affect ligand binding was consistent with these models. Marhefka et al. also noted that the MD-processed structure was superior to structures obtained using less-sophisticated homology modeling procedures.

Wang et al. constructed homology models, refined using molecular dynamics, of four human class I histone deacetylase HDAC isoforms[16]. The comparison between the models and X-ray structures of HDAC8 that became available during their study showed excellent agreement, which validated their approach. The RMSD between the backbone atoms of the model and crystal structure was 2.07 Å. The largest differences between the two were mainly in one of the loops, and in flexibility of the entrance into an internal cavity that was seen in crystal structure and also during the time scale of the MD simulation, although this flexibility was not taken into account in the final configuration of the model.

Virtual screening of a database of drugs against a SARS-associated coronavirus (SARS-CoV), 3C-like proteinase ($3CL^{pro}$) model yielded a cinanserine hit that turned out to be active against SARS-CoV and related human coronavirus 229E[17]. The homology model was optimized using energy minimization[18]. The crystal structure of SARS-CoV $3CL^{pro}$ appeared during the study and, hence, could be used for verification. The RMSD between homology model and X-ray structure was 3.11 Å, while RMSD of the binding pocket was only 0.18 Å. The docking simulations and binding affinity calculations were repeated with the ten best compounds from homology model screening. Calculations with the real structure were in good agreement with the data obtained using the model. Earlier, a comparison was also made between virtual screening of 73 various proteinase inhibitors against the SARS-CoV model and its template, the main proteinase of the transmissible gastroenteritis virus[18]. Most molecules bind to the two structures in a similar way, and the correlation factor *R* between their DOCK scores is 0.65. The RMSD of the binding pocket atoms between the template and the model was 0.18 Å, and overall Cα atom RMSD was 0.34 Å.

Because of the availability of an X-ray structure during the final stage of the study, virtual screening was performed for both the homology model and crystal structure of human 11β-hydroxysteroid dehydrogenase (11βHSD1)[19]. The homology model was energy minimized with cofactor included (cofactor was taken from another template). In a similar way, coordinates of a known inhibitor were included from a third template. RMSD between the final model and real structure was below 2 Å (the authors did not specify what atoms were used to compute that figure). Binding modes between the binders docked into both structures were relatively close. Moreover, the correlation between the binding affinities computed using GlideScore of same ligands docked into both structures had correlation coefficient $R$ = 0.69. Correlation factor $R$ between binding affinities of binders predicted from virtual screening was similar, 0.71. Thus, the binding modes predicted from docking into the homology model and X-ray structure were both structurally and energetically similar. It is worthwhile to note that the reported correlation coefficient is good even when compared to typical correlation coefficients between computed and experimental binding affinities for docking into the real crystal structures[20,21].

The basic conclusions from these papers show that homology models in most cases can give a reasonable enrichment of the binders during the virtual screening, and also can give reasonable binding modes and energies.

## APPROACHES TO REFINEMENT OF HOMOLOGY MODELS

In this section, we report different approaches to homology model refinement and highlight some of the most interesting applications.

### Use of Homology Models Without Further Optimization or With Simple Energy Optimization

In this subsection, we will consider cases where the built homology models were used without a subsequent use of MD. Of course, some optimizations may still be necessary, such as optimization of loop and insertion/deletion regions. It should be mentioned that there is a capability of some homology building programs to optimize the side chains in the presence of a binder, to avoid clashes and improve hydrogen binding networks, and to use constraints during model building. Quality of the model depends on a correct sequence alignment. Also, the results may depend on the sophistication of program that was used to build the homology models. Nevertheless, some examples in the literature show significant success of this relatively simple approach.

Kemp et al.[22] computed the correlation between the experimental $IC_{50}$ and docked scores for 33 compounds docked into a model of Cytochrome P450 2D6, and the computed regression coefficient was $r^2 = 0.61$. The method allowed identification of several novel inhibitors.

Arienti et al.[23] used a homology model of DNA damage control kinase chk2 to facilitate optimization of a novel series of inhibitors discovered via high-throughput screening. Their research led to a highly potent 2-arylbenzimidazole ($IC_{50}$ 15 n*M*). Structure-activity relationship was found to be remarkably consistent with the docking into homology models.

Hillisch et al. investigated two isoforms of estrogen receptors (ER) α and β[24]. The binding pocket of the energy-minimized model of the ligand-binding domain of ERβ differs to that of ERα by two amino acids. This allowed researchers to design highly selective agonists of ERα and ERβ using structure-based drug design. These studies led to insights about the physiological roles of the two isoforms.

Interestingly, researchers at Novartis were able to discover potent inhibitors of CDK1 based on analysis of binding mode to a homologous CDK2 of known nonselective CDK1 and CDK2 inhibitors, and mined the company's compound database for the designed scaffold[25]. This was possible because they took notice of the conservation of ligand-binding residues in the ATP-binding pocket between CDK1 and CDK2.

Sometimes, docking is performed by superimposition of the coordinates or translation of small molecules present in the crystal structure of the template or homologous protein. Warhurst built a homology model of *Plasmodium falciparum* DHFR[26]. The drug molecules were taken from existing PDB structures as scaffolds, changed to obtain derivatives, and optimized inside the binding site using molecular mechanics MM+ force field. Implications for drug resistance were discussed and some directions for further drug development were proposed based on the observations.

A model of rabbit organic cation transporter OCT2, which is responsible for renal secretion of cationic drugs, was built from templates that shared <15% with the target, using energy minimization[27]. The model confirmed mutagenesis studies and, in addition, researchers found a significant correlation ($r^2$ = 0.82) between the $IC_{50}$ values for inhibition of tetraethylammonium transport by 14 different compounds and their calculated $K_d$ values for binding to the homology model.

Using a blind-docking (no predefined binding site) study of a known binder against an energy-minimized homology model of DNase γ, Sunaga et al.[28] discovered an alternative binding site, the DNA trapping site. After screening of a ligand database into the trapping site of the model and experimental test of the best hits, they obtained a good correlation between predicted ΔG of docking into the DNA trapping site and experimental $IC_{50}$ ($R$ = 0.8055). There was no correlation between ΔG and $IC_{50}$ for the active site ($R$ = 0.0346), which indicated that the investigated binders bind to the trapping site.

## Using an Ensemble of Protein Structures

Using an ensemble of protein structures can be useful when there are several alternative receptor structures. In simple cases, docking could be independently or sequentially performed against a few structures, for example, agonist- and antagonist-bound receptor structure, or to alternative homology models.

The FlexE method, developed by Claussen et al., uses an ensemble of several structure variants of a protein for docking[29]. FlexE is based on a united protein description generated from the superimposed structures of the ensemble. For varying parts of the protein, discrete alternative conformations are explicitly taken into account, which can be combinatorially joined to create new valid protein structures. FlexE yielded better results compared to using simply merged lists of docking hits into several receptor variants. In the case of aldose reductase, where the ensemble consisted of one homology model and two X-ray structures, they showed that if just one rigid conformation was used, some inhibitors could be missed.

Schafferhans and Klebe developed a procedure, DRAGHOME[30], which combines information from homology modeling with ligand data used by and derived from 3-D quantitative structure-activity relationships (QSAR). The binding site of a model-built protein is analyzed in terms of putative ligand interaction sites and translated via Gaussian functions into a functional binding-site description represented by physicochemical properties. Ligands to be docked onto these binding-site representations are similarly translated into a description based on Gaussian functions. Using DRAGHOME, thrombin inhibitors were docked onto models of thrombin generated using serine protease templates with 28–40% sequence identity. Each model consisted of an ensemble of ten or more structures. The procedure yielded ligand-binding modes with an average RMS deviation of 1.4 Å, where mostly the near-native solutions ranked best.

## Ligand-Directed Optimization of the Binding Pocket

In many cases, the docking of a ligand to a binding pocket is, solely, the direct target of the virtual screening studies. Hence, it could be enough to optimize the shape of the binding pocket so that the binding modes of one or several ligands are correctly represented. Moreover, conventional optimizations including MD, cannot correct alignment errors or reproduce helical shifts and distortions, or correctly model long irregular loops[31]. Hence, model refinement requires incorporation of additional information, including mutagenesis information or knowledge/hypothesis about the binding mode of a ligand. Similar to mutagenesis in spirit is a structure-based screening method using NMR data[32]. In this method, one looks at the appearance/disappearance of NMR interaction signals when different ligands are bound, and this information can be combined with structural studies.

Ligand presence can be taken into account via side chain rotamers. In an extensive study by Rockey and Elcock[33], for seven protein kinase inhibitors, relative binding affinities with a panel of ~20 protein kinases were computed and compared with experimental data. When building homology models, no attempt was made to model insertions relative to crystal structure, and the residues outside of catalytic (ATP-binding domain) were omitted. The side chains were optimized using SCWRL[34] in the presence of the ligand. Their ligand-receptor affinity computation program SCR was able to give an excellent reproduction of the experimental trends and, importantly, was capable of identifying the targets of inhibitors even when they belonged to different kinase families. For the previous test, it was assumed that inhibitors dock in the same orientation for all kinases. It also turned out that results became worse if the ligands were docked independently and then scored. Relaxation of the inhibitor and receptor complex in solvent using GROMACS[35] did not improve the results. These results were explained by the fact that using fixed orientation of ligands across all kinases better highlighted identical interactions between ligand and receptor. Finally, three inhibitors were screened against a set of 493 kinases and the list of predicted binding receptors was enriched with true experimental targets.

A good example of guiding by bound-ligand approach is a study by Johnson et al. of anti-*Shigella flexneri* Y monoclonal antibody (mAb) SYA/J6[36]. A diverse library of protein models was created and then screened by docking with ligands of known conformation. A significant correlation was observed between the docked ligand energies and correctness of conformation for the hypervariable loop SDR H3, as well as orientation of $V_H$ and $V_L$ domains with respect to each other. This means that the more accurate protein models formed higher-quality docked complexes and the quality of the fit could be used to select the best models.

Bissantz et al. performed a study of screening against several G-protein coupled receptors (GPCRs)[37]. After a 3-D structure of protein was built, antagonist or agonist molecules were positioned inside the binding pocket and energy minimized. The homology models were used to screen 3-D databases using three different docking programs (Dock, FlexX, Gold) in combination with various scoring functions. The built models enabled them to retrieve known antagonists from ligand databases. To generate receptor models better suited for agonist screening, they developed a knowledge- and pharmacophore-based modeling procedure that might partly simulate the conformational changes occurring in the active site during receptor activation. The same group later developed a GPCRmod package aimed at the high-throughput modeling of GPCRs, built a library of 277 GPCR targets, and used inverse screening (one molecule is docked against many receptors) to validate their models by recovering the receptor of a selective GPCR antagonist as well as the receptors of a promiscuous antagonist among top scorers [38]. The structures were refined using energy minimization.

Ashton et al. reviewed different approaches to virtual screening of GPCRs[39]. In a case study, they were able to retrieve 50% of the binders in the first 10% of a compound database screened against vasopressin V1a receptor using a procedure similar to that used by Bissantz et al.[37]

Klebe's group developed the MOBILE approach[40], which combines receptor-ensemble and ligand-directed optimization approaches. In a first step, ligands are docked into an averaged ensemble of crude homology models of the target protein. In the next step, improved homology models are generated, considering explicitly the previously placed ligands by defining restraints between protein and ligand atoms. These restraints are expressed in terms of knowledge-based, distance-dependent pair potentials, which were compiled from crystallographically determined protein-ligand complexes. Subsequently, the most favorable models are selected by ranking the interactions between the ligands and the generated pockets using these potentials. Final models are obtained by selecting the best-ranked, side-chain conformers from various models, followed by an energy optimization of the entire complex. The approach was applied on factor Xa and aldose reductase homology models. Including ligand information in the homology model yielded structures better suited for structure-based drug design. The MOBILE approach was applied to a neurokinin-1 receptor belonging to the GPCR family[41]. For the construction of the NK1 receptor, antagonist CP-96345 was used to restrain the modeling. The quality of the obtained model was validated by probing its ability to accommodate additional known NK1 antagonists from structurally diverse classes. On the basis of the generated model and on the analysis of known NK1 antagonists, a pharmacophore model was deduced, which subsequently guided the 2-D and 3-D database search. As a following step, the remaining hits were docked into the modeled binding pocket of the NK1 receptor. Finally, seven compounds were selected for biochemical testing, among which one showed affinity in the submicromolar range.

Evers et al. used a modified, ligand-directed MOBILE approach to generate a model of α1A receptor[42]. Notably, there is no sequence identity in the binding site between the template bovine rhodopsin and the target. After a hierarchical virtual screening procedure guided by 2-D filters and 3-D pharmacophore models, ca. 23,000 selected compounds were docked into the model using GOLD[43] and scored with PMF[44]. From 80 top-ranked diverse compounds, 37 compounds had $K_i$ values better than 10 μ*M*, and the most active bound with $K_i$ = 1.4 n*M*.

In a recent application that used the MOBILE approach, Evers et al. built homology models of several biogenic, amine-binding GPCRs (α1A, 5HT2A, D2, and M1 receptors) and compared performance of QSAR and docking-based virtual screening approaches when retrieving known antagonists from virtual libraries[45]. They found that the QSAR techniques (ligand-based pharmacophore, Feature-Tree, 2-D QSAR) provided better enrichment factors compared to molecular docking. However, they also point out that it is unclear how much docking results would improve if better-quality structures were used for docking. In addition, docking is most valuable in cases where either limited or no ligand information is available, or for discovering of novel ligand classes/binding modes.

## Partially Optimized Structures

Takami and coworkers built a homology model of Rho kinase based on protein kinase A (cAPK)[46]. The side chains were kept as close as possible to the template. After building the model, energy minimization was performed, keeping backbone atoms fixed, except for the modeled loops. Based on hits of high-throughput screening against the homology model, a synthetically feasible virtual library was designed. With the aid of docking simulation, possible active structures as well as possible inactive structures from the virtual library were synthesized and submitted for biological assessments. The obtained results proved that their model was a reliable one.

In a study by Gessner et al.[47], human EAG1 and EAG2 potassium channel models were energy minimized using weak constraints on protein backbone because minimization was performed in the absence of solvent. After the ligands were placed in the binding site so that they have minimal steric clashes, the docked complexes were further energy minimized in two modes: keeping the protein rigid (rigid docking) or allowing for some conformational readjustment with positional restraints on all protein atoms (semiflexible docking). Only with the semiflexible docking approach were they able to explain qualitatively the molecular mechanisms underlying the differences in efficiency of channel blocking by clofilium, quinidine, and tetrabutylammonium.

## Full Optimization of Ligand and Receptor using MD or Related Procedures

The approach that applies MD for refinement of the homology model is presently the most used. The consensus procedure is building the homology model, refining the structure using MD, especially for the systems with low identity between the target and template, such as GPCRs, and subsequently validating the structure. As noted above, the MD approach sometimes can fail, for example, to correct alignment errors or reproduce helical shifts, or model long irregular loops[31]; hence, an approach that uses additional information, such as mutagenesis studies, is needed.

Several examples emphasize the advantage of the MD-optimized structure over simple energy optimization. It was mentioned earlier that Marhefka et al. found that the MD-optimized model of human androgen receptor was superior to a simple optimization-refined structure[15]. In a work by von Langen et al.[48], a glucocorticoid receptor, ligand-binding domain was built with an energy-optimization refinement, and docking of several steroids into its binding site yielded the wrong binding affinity rank for cortisol with respect to other steroids because of an incorrect hydrogen-bonding pattern. Averaged structures were generated for 4 nsec MD simulations with each complex. The scoring with the Molecular Mechanics Poisson-Boltzmann Surface Area (MM/PBSA)[49] method allowed them to discriminate correctly between strongly and weakly bound compounds. FlexX[50] also correctly identified cortisol as the ligand with the highest binding affinity. The authors note that even docking cortisol into a structure optimized using a different ligand is better than the initial unrefined model.

Zabell et al. built a homology model of human low-molecular-weight protein tyrosine phosphatase isoform B (HCPTP-B)[51]. A comparison of the two isoform structures indicated the possibility of developing isoform-specific inhibitors of HCPTP. MD simulations with CHARMM[52] have been used to study the binding modes of the known adenine effector and phosphate in the active site of both isoforms. This analysis led to the design of the initial lead compound based on an azaindole ring moiety. The simulation studies highlighted the need for a phosphonate group on the indole and provided insight into inhibitor-binding modes. Compounds with varying degrees of structural similarity to the azaindole were synthesized and tested for inhibition with each isoform. Two compounds were experimentally found to have submillimolar inhibition.

Sterol 14α-demethylase (CYP51) is the only CYP family in the cytochrome P450 gene superfamily found in both eukaryotes and prokaryotes[53]. Fungal CYP51 is one of the known major targets of azole antifungal agents. Therapeutic side effects of these antifungals are based on interactions of the azoles with the human analogue enzyme. The study by Rupp et al. describes a comparison of homology models of human and *Candida albicans* CYP51, constructed from *Mycobacterium tuberculosis* CYP51 X-ray structure[54]. The binding mode of the azole ketoconazole is investigated using MD simulations with the GROMACS force field. The usage of special parameters for the iron-azole complex binding is necessary to obtain the correct complex geometry in the active site of the enzyme models. Based on the MD simulations, it was possible to explain the enantioselectivity of the human enzyme and also to predict the binding mode of the isomers of ketoconazole in the active site of the fungal model.

Moran et al. separately built two nucleotide-binding domain dimeric subunits of cystic fibrosis transmembrane conductance regulator (CFTR)[55]. In order to remove clashes between the subunits, the MD was used to equilibrate the dimer at 300 K. The ATP molecules from the template were included in the optimization. The correlation between the experimental apparent dissociation constant of the activators and the computed dissociation-free energy was used to determine the putative binding site. Out of three activator binding site candidates, site 2, which had Pearson correlation coefficient $r$ = 0.83, appeared to be the likely binding site. The other sites had very poor correlations of –0.07 and –0.46.

Bjelic and Åqvist used a combination of homology modeling, automated docking searches, and MD/reaction-free energy profile simulations to predict the structure of *P. falciparum* histoaspartic protease, conformation of bound substrate, catalytic mechanism, and rate of the peptide cleavage reaction[56]. They found that the computational tools are sufficiently reliable, both for identifying substrate binding modes and for distinguishing between different possible reaction mechanisms. Their calculated catalytic rate constant of about 0.1 $sec^{-1}$ for a hexapeptide substrate derived from the R chain of human hemoglobin is in excellent agreement with experimental kinetic data for a similar peptide fragment.

Avery's research group performed screenings against several homology models to improve selectivity. Virtual screening of the ChemBridge database of compounds was performed against models of malarial cysteine proteases falcipain-2 and falcipain-3. A total of 24 diverse inhibitors were identified, of which 12 compounds appeared to be dual inhibitors of falcipain-2 and falcipain-3. Four compounds showed inhibition of both the malarial cysteine proteases as well as *Leishmania donovani* cysteine protease[57]. The models of falcipains were refined and validated by docking of known inhibitors and applying MD simulation[58,59].

Muegge and Enyedy performed docking into a ErbB2 kinase model refined using MD simulation[60]. After screening of a database, 144 compounds were chosen for biological testing, ten of them showed inhibitory activity against ErbB2, and one compound appeared to be a selective and potent irreversible inhibitor with IC50 1.5 μ*M*, which was unexpected since screening focused on noncovalently bound inhibitors.

Schapira et al. performed compound library screening against several nuclear receptors, including a computer model of glucocorticoid receptor (GR), and the three known GR agonists were found among the top 1% best compounds[61]. The 3-D model of the GR ligand-binding domain used for screening was built by homology to the crystal structure of the androgen receptor, a template sharing 53.8% sequence identity. The target sequence was aligned on the 3-D template, and the energy of the system was minimized by a Monte Carlo simulation through a series of random global moves and local gradient minimizations in the internal coordinates space. A single rotameric state was used for screening.

MD can be applied to the receptor structure and optimized prior to docking. In the docking studies, this receptor structure can be treated as rigid. Alternatively, receptor flexibility could be taken into account during docking. Carlson and McCammon[62] analyzed an approach where an ensemble of structures resulting from simulation is used for docking, implicitly including receptor flexibility. Sine et al. built a homology model of acetylcholine receptor (AChR)–binding domain, belonging to a superfamily of pentameric ligand-gated ion channels[63]. An ensemble of 1000 frames from MD simulation was used for docking. A similar procedure was also applied to the template for validation. The resulting ligand orientations were clustered into groups. The distinctly different docked orientations for *d*-tubocurarine and metocurine were confirmed using mutagenesis results

Moran et al. used MD on a complex receptor system consisting of a homology model of CLC-0 $Cl^{-}$ channel, phospholipid bilayer, and a shell of water[64]. During MD simulations, a constant force was applied to a channel-blocking molecule, *p*-chlorophenoxyacetic acid (CPA), pushing it forward to the center of the channel. This allowed them to outline a possible intrachannel pathway of CPA, and to describe qualitatively the binding sites and energy barriers along this pathway. The obtained results were consistent with experimental data.

## CONCLUSIONS

In spite of the boom in experimental determination of protein structures, only about 7500 different structures have been solved, and we saw a rapid increase in the use of homology models for structure-based exploration of protein properties. We can find many examples that show the validity of using homology models for structure-based drug design. The models are able to enrich screening results with known binders, predict correct binding geometries and affinities, as well as give insight into functional mechanisms of proteins. However, the number of experimentally determined 3-D structures of proteins is low to provide meaningful templates to build models for the whole protein universe. Depending on the similarity between the template and the target, a variety of methods can be used to optimize the model. If the degree of similarity between the target and template is relatively high, simple refinement methods could be sufficient. MD simulation is probably the most advanced method available for model refinement, especially if the identity is low, can largely take into account receptor flexibility and advances in force fields, and parameterization and computer algorithms will allow us to construct reliable models with sequence identities just above 20% in the near future. But, we will certainly need to incorporate data from other sources to supplement the structural data available if we want build homology models below this threshold. It will be done more frequently with data coming from mutagenesis, ligand binding, thermodynamics, and functional and metabolic studies. This will become an ordinary part of the homology building protocol (probably becoming an automated procedure), presumably via structural or energetic constraints during the model building and refinement steps. We can expect that protein folding, docking of the binder, and homology model refinement, including the use of molecular dynamics, eventually will be integrated into an efficient procedure.

## ACKNOWLEDGMENT

One of the authors (V.K.) is grateful for a support from Fundação para a Ciência e a Tecnologia of Portugal (grant SFRH/BPD/20716/2004).

## SUPPLEMENTARY MATERIAL

A table containing a list of types of receptors for which homology models were built and used for docking, together with a list of 273 references, is available from the website of this Journal.
